# Dr. Reid on Medical Botany

**Published:** 1832-10-01

**Authors:** 


Outlines of Medical Botany, comprising Vegetable Anatomy and Physiology, the Characters and Properties of the Natural Orders of Plants, an Explanation of the Linnæan System of Classification, and Tables of Medicinal Plants, arranged in their Linnæan and Natural Orders. By *Hugo Reid*, Member of the Royal Physical Society and of the Society of Arts, Extraordinary Member, and lately President, of the Hunterian Medical Society of Edinburgh. 12mo, pp. 390. Edinburgh, 1832.

At all times, but especially in the present season of the year, when Nature is clothed in her loveliest garb, and invites the most thoughtless and ignorant to the contemplation of her treasures, it is our delight to study Botany; to leave for a time the filth of the dissecting room, the closeness of the laboratory, or the fatigues of actual practice, and roam at liberty over hill and dale, admiring the beauties and wonders of the vegetable world, gathering the choicest specimens, and afterwards carefully preparing them for the herbarium. Were the medical profession sufficiently impressed with the interest, and, we may add, importance of botanical science; could every young member of it, before he engages in the turmoils and anxieties of busy life, taste of the pleasing delights which its votaries enjoy, and could they, while yet at the lecture-room, foresee the varied situations in which they might afterwards be placed, situations in which the medical man is continually made the referee upon any dispute, or regarded as the expounder of any difficulty, we doubt not that much more attention would be paid to the acquisition of the knowledge of plants than has hitherto been; and no argument or persuasion be necessary to prompt and stimulate him to its study and pursuit.

We are well aware that a very common opinion prevailing is that this department of natural history is trifling and unimportant; that the objects of its research, though beautiful, are to a very limited extent of much utility, especially in the present day, when we have a sufficient number of officinal herbs, if we only knew how to use them; that the details of classification and arrangement are most absurdly tedious and perplexing, and require so much time, as to withdraw the attention from objects of more direct practical consequence. That difficulties must be encountered, and overcome; that uncertainties not unfrequently arise, and that very considerable leisure and patience must be devoted to the study, at least when it is first entered upon, we do not deny; but these are objections, if objections they can be called, not peculiar to botany, but to every branch of science; the threshhold is seldom very inviting; the steps which lead up to the porch may be many and wearisome to mount, and when we reach the vestibule we may feel ourselves lost, as it were, in the amazing extent, and vast dominions of the temple; but every advance we make will render our future efforts more easy, and what would have been fatiguing at first, will soon be felt as light and pleasant; for we gain strength as we go on, and the capacities of our minds become more exalted and amplified. We doubt not but that the mighty mind of Linnæus, when engaged in his great enterprise of classifying the infinitude of Nature's works, was much less oppressed with his labours than the young student when he first endeavours to ascertain the genus of a plant. But we shall revert to this subject, and shew that botany, if properly studied, is of all sciences one of the most easy of acquisition, as well as one of the most delightful in its rewards; and in the mean time we shall take our stand in defence of this favourite pursuit on a different and more worthy ground, and we shall appeal to the better and more generous principles of youthful aspirants to medical fame; we shall view the subject, not merely in regard to the difficulties which may attend its acquisition, or to the immediate and visibly obvious importance of its details, but as it is calculated to improve, and embellish the minds of our professional brethren. For, alas! in the present day, and especially in this great metropolis, the name and character of a surgeon and physician are sadly degraded; too often are they associated with the ideas of chicanery, and subserviency; and little wonder is it when we consider that such a large portion are on a level with petty traders, and sneaking shop-keepers. Medicine is not followed as a science, but as an occupation; and the hopes and wishes of its votaries are limited to the base and illiberal calculations of profit and of gain. As long as the present system of medical practice exists, we can hope for little improvement; and we are not of such Utopian extravagance, as to imagine that we can in any measure effect a change in the present old established generation of medical men, but we are anxious to contribute our support to the bringing in of a better state of things, when our profession will be studied and pursued as a branch of useful science, and as the channel of the choicest blessings of benevolence and sensibility. It must be divorced from all connexion of traffic and trade; its members, if they have any spark of gentlemanly feeling, must separate themselves from any such unions as that of compounding and dispensing, and retailing drugs; they must have a pride above such humble, and lowly occupations; and their thoughts must be directed to the acquisition of liberal knowledge, and to the diffusion of its blessings to those around them. Now we do not know a better method to effect this, than to inspire every young medical man with a taste for some of the branches of natural history, which, independent of its many direct associations with medicine in the departments of comparative anatomy, materia medica, and toxicology, cannot fail to imbue the minds of its students with a love of science, for science's sake; and if one department is more inviting than another, that one we think is botany.

There is a healthfulness, and gaiety of body and of spirits in its pursuit; its objects are so wonderfully beautiful and curious, and the facility of preserving them is so pleasing, that apart from their utility and importance in husbandry, gardening, and medicine, it appears to us that they only require to be known, to excite a zeal and enthusiasm in their favor. What can be more interesting, and also more worthy of an intelligent mind, than to find amusement and instruction in every walk? what more soothing and quieting to the feelings, after the vexatious annoyances and disappointments of a day's labour, than to contemplate Nature in its gentlest and fairest forms? Those, indeed, who are immersed in the walls of a large city are in a great measure shut out from such pursuits; but even they have opportunities of visiting gardens, and hot-houses, and conservatories; and with an herbarium at home, they can almost live over again those happy days when they were engaged in collecting its specimens. How few, however, of the medical republic are these, in comparison with the sojourners in the country, or the wanderers by sea and land in foreign climates; and we know from our own experience what a rich and constant theme of pleasure and delight botany may become to them. He who has visited the shores of a tropical climate, and has gazed on the wonders of tropical vegetation, and yet has not felt within him a wish to hold, as it were, converse with the mute beings which surround him, in all their luxuriance of beauty, and gorgeousness of profusion, must have a soul dead to every love of science, and is not worthy of being her votary, in the career of a liberal and ennobling profession.

We will not deny that such a man may be a useful practical surgeon, or physician; he may be able to amputate a limb, or dress a sore, or prescribe for an inflammation as well, and as expert as he who adds a taste for botany, or zoology, or mineralogy, to his professional acquirements; but the reverse of the position is equally true, and will not we hope be disputed; we mean, that the student of science and lover of Nature may be as thoroughly acquainted with the details of practice, and as intimately conversant with the duties of the hospital, or sick-room, as he who limits his ideas within such a sphere; and we shall go a step further, and boldly assert that almost all the great improvements in physic and surgery have emanated from scientific men. Where can you find a better physician than the accomplished Mead; or what name is worthy of being joined with Hunter's?

It is not so much from the mere acquisition of the facts of knowledge; it is not only from being able to tell all the minutiæ of animal structure, from the anatomy of man to that of the simplest worm; or to recognise and name every plant from the lofty palm to the microscopic moss, that he whose mind is thus enlightened has a higher chance of adorning and improving his profession; what is more important is that besides having a well-stored memory, he has been accustomed to habits of patient and assiduous research; he has been taught to examine facts and objects for himself, to compare them together, to note in what they resemble, and in what they differ from each other, and thus he acquires that sifting frame of mind which is so well calculated to observe the phenomena of health and disease, to discard all prejudices, and all opinions which are not substantiated by an appeal to experiment and observation, and to base the general rules of his practice on a most cautious and severe induction. We cannot, therefore, too earnestly recommend, as an accessory or collateral branch of knowledge of high importance, botany in its various details. Naval and military surgeons have frequently much spare time, and placed as they are so frequently in situations most favourable to the prosecution of the study, that leisure might be thus most agreeably and usefully employed, and the character of the medical profession be most materially exalted and respected. We shall not urge this topic any further; but shall now briefly explain the manner in which a competent knowledge of plants may be easily obtained. The ordinary routine of attending a course of lectures, and of a few specimens being put into the hands of the student in illustration of these, will avail little; it will enable the student to discourse sufficiently on opinions and theories; to understand the teasing vocabulary of names, and even to ascertain a few prominent facts, such as the class and order of a specimen, provided the flower be present; but such knowledge, disjoined from a practical acquaintance with plants in their native localities, is only ornamental, and savours often too of book pedantry. A week or two's reading in the closet will be sufficient for this part of the subject; but it is only in the fields, and from Nature's own book, that we can expect to become botanists. With our box slung across the shoulders, and our Compendium in our hands, we must start with as much glee as the huntsman for the chase. But ours is the more exalted, and in course of time becomes by far the more agreeable sport; ours is not to inflict pain, or to murder feeling life, but to search after, and to look into the choicest and sweetest productions of the Creator's hand.

There is altogether a something so cheerful, so soothing, and calm and intellectual in the study of the external world, so well fitted to withdraw the mind from the tumult of passions, from the corrosion of cares, to exalt it above the lust of money and of wealth, to inspire it with a love of knowledge for the sake of knowledge herself, and thus to throw, as it were, around it, an intellectual mantle, that he is little to be envied, who, although surrounded with affluence and power, is shut out from such high enjoyments by wilfulness or by ignorance. Let us consider too, how easily and how agreeably are such enjoyments purchased; the very pursuit of them is full of health-giving spirits, and so abundant are they that all may be satisfied, and yet no satiety be felt by any: we have only to wish to possess them, and to add to that wish a little willingness for exertion, and soon we may acquire a considerable knowledge of practical botany. As the difficulties which the young student encounters at first, from the immense vocabulary of designating words—from the inapplicability of many of these—from the unsteady characters of some plants, depending upon age, locality, and so forth, we consider by far the best plan of teaching botany to be, what may sufficiently appositely be termed the Hamiltonian system; the principle of which is to introduce the pupil at once into the "medias res," and to convey the knowledge of the grammar and philosophy of the science to him as he proceeds: thus every plant which he collects is interesting and instructive, not only as an addition to his herbarium, but as the type of a term of nomenclature, or an illustration of a physiological truth. Our advice to our young medical brethren is at once to betake themselves to the practice of botany, under the guidance of an experienced teacher, who will accompany them in their journeys and excursions: we know the advantages of this method from our own experience, and have much pleasure in availing ourselves of the present opportunity of recommending to the alumni of the Edinburgh Alma Mater, the name of Mr. Lloyd, author of botanical terminology, a work well calculated to explain the numerous terms which are used in the description of plants.

After these preliminary observations, we shall proceed rapidly to review the work before us, which is highly creditable to our young friend, and calculated we think soon to supersede Smith's Elements, or any other manual on the subject. We have carefully examined Mr. Reid's Outlines, and cordially approve of them; indeed it is decidedly the best introductory book upon the subject. The descriptions are clearly and concisely detailed, and very well illustrated by neat wood-cuts, introduced separately into the pages instead of being collected together at the end of the volume. This is by far the better plan, as we thus have the benefit of the verbal and pictorial delineations at the same time. The work consists of two parts; the one devoted to an account of the general and special anatomy and physiology of plants; the other to the arrangement, or classification of them. We are tempted to select a few quotations to shew the matter and manner of the contents.

"The only phenomena in the physiology of plants which have any appearance of being connected with a nervous system are those which are more correctly said to be caused by their irritability. Examples of this property in plants are within every one's observation. The turning of leaves and flowers to the light, even though repeatedly put out of their natural position, and the opening and closing of flowers, are well known. The stamens of the Rock-rose *(Helienthemum)* and of the Barberry *(Berberis)* possess the peculiar property of bending when irritated by any sharp body. The leaves of the *Hedysarum gyrans*, a plant found in India, are almost constantly in a state of motion. The leaf of the *Dionea muscipula* (Venus' Fly-trap) is provided with bristles, and, when irritated by an insect, it becomes folded and crushes the animal. The most remarkable instance of irritability in vegetables is afforded by the sensitive plant *(Mimosa pudica)*; when touched or agitated by the wind, the leaflets become erect, and closely applied to each other and to the stalk, but they gradually resume their original position when the stimulus is withdrawn. Besides these, there are many other instances which it would be unnecessary to enumerate here: indeed, it is not improbable that it is by a kind of irritability, or contractile power, that the rootlets are enabled to absorb the juices, and the vessels to propel their contents. It has also been lately ascertained that vegetables are acted on by those poisons which are supposed to affect animals through the medium of the nervous system, as hydrocyanic acid. The effect generally was to produce a kind of contractile action, and diminish the sensibility of the plant to the usual stimuli." 8.

The description of the functions of the root is succinctly and cleverly given.

"Thus it will be observed that perennial plants, which live during many seasons, have either a bulb, a tubercle, or a woody subterraneous stem, which preserves the vital principle during the suspension of vegetation, and contains a store of nutritious matter for the early growth of the new plant. A mere bundle of fibres would be unable to survive excessive cold or much moisture, could not contain a store of ready formed vegetable matter, and would not be adapted to convert readily the material drawn from the soil into proper nutritious matter. In every instance of the growth of a new plant, whether an entire plant from a seed, bulb, or bud, or herbage and flowers from a perennial root, there must be a stock of proper food ready for the young plant.

Almost all plants are provided with roots, except several of the lower orders, which, vegetating in water or on its surface, absorb nutritious matter at every point. Some plants which have roots float loosely in water, and are not fixed to any thing, as Duckweed *(Lemna)*. Aquatic plants, however, have generally two roots; one is buried in the earth and fixes the plant; and the other floats freely in the water, as Buckbean *(Menyanthes)*, and Water Lily (*Nymphœa*). The Sea-weed tribe *(Algœa)* are in general fixed to rocks, from which they can draw no nutritious matter.

PARASITICAL plants are those which insert their roots in other plants, and draw from them their nourishment: Broomrape *(Orobanche)*, Dodder (*Cuscuta*), Misseltoe (*Viscum album*).

Branches also possess the property of throwing out rootlets (121.): if a branch be placed in the earth, or surrounded with earth on the tree, an incision being made in the bark, it will emit rootlets from its sides, and become, if properly treated, an entire and independent tree; hence the propagation of plants by slips and by layers. Many of the Grasses, as Indian Corn (*Zea mays*); and the Sugar Cane (*Saccharum officinarum*), emit from the knots on the stem, when these parts are surrounded with moist earth, rootlets capable of producing new plants. In this way the Sugar Cane is propagated." 23.

It is curious to remark the analogy which exists between various parts of a vegetable and the corresponding textures in animals:—read the account given of the functions of the epidermis.

"The cuticle is supposed to protect the parts underneath from the too direct action of air and water, and to prevent too great evaporation of the fluids. It affords little protection from the action of heat or cold, except when covered by a thick hair or wool, as in the Great Mullein. On the trunks of the Fir, the Plane, the Oak, and other trees, the office of the cuticle seems to be performed by dead layers of bark or of herbaceous integument, which are pushed outwards, having performed the functions for which they were made.

In forest trees, and in the larger shrubs, the bodies of which are firm and of a strong texture, it is a part of little importance; but in the reeds, the grasses, canes, and the plants having hollow stalks, it is of great use, and is exceedingly strong; and, by the microscope, seems composed of a kind of glassy network, which is principally siliceous earth. This is the case in Wheat, in the Oat, in different species of Equisetum, and, above all, in the Rattan, the epidermis of which contains a sufficient quantity of flint to give light when struck by steel. The siliceous epidermis serves as a support, protects the bark from the action of insects, and seems to perform a part in the economy of these feeble vegetable tribes, similar to that performed in the animal kingdom by the shell of the crustaceous insects. I have ascertained by experiment that siliceous earth generally exists in the epidermis of the hollow plants*.' The Bamboo (*Arundo bambos*) contains much silica, called "tabasheer." 31.

The subject of the nutrition of vegetables is full of most curious and interesting research, from the absorption of the food from the earth and atmosphere through the radicles and leaves, to the perfection of the sap, and the formation of its varied products. We give the following extract as it has reference to an hypothesis of a French philosopher, which has been applied to the explanation of animal, as well as of vegetable physiology.

"The cause of the ascent of the sap is not well ascertained. Some have ascribed it to the influence of heat, others to capillary attraction. It seems to depend upon some principle not connected with the vital properties of plants: it has been found that if a root full of moisture be surrounded with very dry earth, the fluid will pass from the root into the latter; and the known effects of heat would seem to corroborate this opinion. It has been ascribed to a vital irritability in the vessels, to the influence of electricity, and by M. Dutrochet to *Endosmose*. This celebrated physiologist found that mucilaginous fluids have the property of overcoming the power of gravity, and ascending in small tubes with a force capable even of overcoming the pressure of the atmosphere, when in contact with membrane or other organized tissue closing the bottom of the tube; the fluid gradually works its way through the membrane or other tissue, and ascends to a great height in the tube. To this phenomenon he gave the name of Endosmose, and applied it to the explanation of the ascent of the sap vegetables.

The sap, when it arrives at the leaves, is deprived of its watery part by exhalation or evaporation, this being dissolved in the atmosphere when it comes into contact with it. This takes place principally under the influence of light and heat. Hot and dry weather greatly facilitates this operation, as Hales ascertained by experiments on the Sunflower (*Helianthus annuus*), which was found in such weather to transpire thirty ounces daily, being one-half more than its average quantity. The watery part of the sap, having performed its office of dissolving the solid matter necessary for the nutrition of the plant, thus rendering it fit to be absorbed by the spongioles, is discharged as of no further use." 60.

Again, the wonderful process of fecundation and reproduction must arrest the attention of every one. Nowhere is the adaptation of structure to function more beautifully illustrated.

"The pollen is conveyed to the stigma in various ways. In many hermaphrodite plants (206), the stamens are longer than the pistil; and the pollen, when it escapes from the anther, naturally falls on the stigma. In such cases, as Linnæus remarked, the flower is generally erect. When the stamens are shorter than the pistil, the flower frequently hangs downwards or droops, thus still enabling the pollen to fall on the stigma when it escapes. When the stamens are short, and the flower erect, there is frequently a nectary at the bottom of it, which attracts insects in search of honey; these become covered with pollen, which falls from them on the stigma as they fly out. The stamens of many plants are endowed with a certain degree of irritability, by which they are enabled to bend towards the stigma and deposit their pollen, returning afterwards to their usual position.

In monœcious (207) plants, the male flowers are frequently situated on the upper part, so that the pollen falls on the stigma. In these, and in diœcious (207) plants, the wind is perhaps the chief agent in conveying the pollen, which

---

* Sir Humphrey Davy's Agricultural Chemistry.

is a light powder, from the male to the female flowers; and butterflies and other insects, which fly from flower to flower, also carry and deposit the pollen. In Persia, the female or fertile Palms chiefly are cultivated, and at the flowering season branches of Wild Palms with male flowers are gathered and shaken over the fertile flowers. This was practised long before the theory of the operation was known.

When fecundation has been effected, the stamens and floral envelope, and also the style and stigma, are of no further use. They generally wither and fall off, though occasionally one or more are persistent. The calyx frequently forms a covering for the ovary, as in those cases where the ovary is inferior, as in the apple; the stigma frequently remains as in the Poppy. The ovary, of course, constantly remains and forms the future pericarp or covering of the seed (356), while the ovule becomes the seed." 99.

It is well known that the propagation of plants may be effected in several ways, besides that of placing the seed in the ground, by buds, and bulbs; by layers, runners, slips, and by grafting; akin to some of these processes are the phenomena of reproduction in the lower orders of zoophytes, the different portions of their bodies being capable of constituting perfect and entire animals. The following observations we consider worthy of insertion.

"Plants are propagated by buds in four different ways: 1. by means of the bulbs which grow at the base of the scales in the bulbous root, as in the Snowdrop or Lily; these bulbs are soon detached from the parent bulb, and become independent plants: 2. by means of the bulbils which grow upon the stem in the axilla of the leaves, as in the Coral-root (*Dentaria bulbifera*), and in the Orange Lily (*Lilium bulbiferum*); or in the place of the flowers, as in the Mountain Garlic (*Allium carinatum*): 3. by means of the buds or small bulbs which grow at the margins of the leaves in the *Bryophyllum*, and the Bog Orchis (*Malaxis paludosa*): and 4. by means of the minute buds or eyes found in the tubercles of various plants, as the Potatoe (*Solanum tuberosum*).

All these buds resemble seeds in this, that when detached from the parent, and placed in the earth, they produce new plants. They differ from seeds, in not being formed by sexual organs; in not being able to preserve their vitality for such a length of time; in not having distinct parts, such as radicle, gemmule, and cotyledons, being merely extensions of the substance of the parent; and in always producing the same variety. Hence the advantage of propagating the Potato by buds; we have found a variety well adapted for use as an article of food, and we can ensure its reproduction. If grown from a seed, a very different variety might be produced, which would not have the same nutritious properties.

Alpine Meadow-grass (*Poa alpina*), and Viviparous Alpine Bistort (*Polygonum viviparum*), frequently bear little buds in their spikes. All plants which increase by buds or bulbs produce few seeds, and are called *viviparous*." 120.

And again:—

"These processes for the propagation of plants, are, in many cases, preferred to multiplying by seed. Propagation by slips or layers always produces the same variety as that from which the slip is taken; so that, if we have a plant which produces good fruit, by propagation in either of these modes, several may be raised bearing fruit equally good. The tree is always more speedy in bearing fruit when formed in this way, than when grown from a seed.

It is a remarkable fact, and one which is turned to good account in the cultivation of fruit trees, that, when the tree is raised in this way, the number of seeds in the fruit is almost always less than when produced from a seed, so that more of the juices and strength of the plant are expended in perfecting this fruit. The Vine, when raised by seed, has four seeds in each grape; but frequently only two, when propagated by layers. The Sugar-cane, which is propagated in a nearly similar manner (64), bears no seed at all, but the other parts of the plant are richly developed.

Thus, by a singular control over their mode of growth, which is also exhibited in the case of viviparous plants, vegetables are enabled to adapt the number of seeds to the demand for them—the demand depending on the number of other sources for propagation which they may possess." 122.

The utility of propagating plants by grafting is thus succinctly explained:

"By grafting we are enabled to accelerate considerably the fructification of various trees, improve much the quality of the fruit, and preserve and multiply particular varieties of trees which may be deemed valuable. An Apple-tree does not in general produce fruit till it is ten years of age; but if a scion of a seedling tree be grafted on one that has already borne fruit, it will bear fruit in the third or fourth year. 'Suppose two acorns of a new species of oak received from a distant country; sow both, and after they have grown one or two years, cut one of them over, and graft the part cut off on a common oak of five or six years' growth; the consequence will be, that the whole nourishment of this young tree of five years' growth being directed towards nourishing the scion of one or two years, it will grow much faster, and consequently arrive at perfection much sooner, than its fellow, or its own root left in the ground. A French author found the advantage of this practice, in the case of a new species of ash, to be as five to one in point of height.'—*Loudon's Encyclopædia of Gardening.* The quality of the fruit is improved by the non-development of many of the seeds of grafted plants, which enables them to expend more of their energies in enriching the fruit. By grafting, we can supply a branch of an old tree with a plentiful quantity of sap, more healthy and nourishing than it could get from the tree on which it grew, and thus for a time it is more vigorous, and produces richer fruit. 'As the graft is merely an extension of the parent plant from which the scion came, and not properly speaking a new individual, so it is found to be the best method of propagating approved varieties of fruit-trees, without any danger of altering the quality of the fruit.'—'Till lately, grafting was confined to the ligneous plants, but it is now successfully practised on the roots and shoots of herbaceous vegetables; and the Dahlia is grafted by the root; the Melon on the Gourd; the Love-apple on the Potato; the Cauliflower on the Cabbage, &c. by the shoot."—*Loudon.*" 124.

The author next relates the difficult details of vegetable chemistry; but to this part of the subject we have not space to devote. We therefore proceed to the second great division of the work, that of systematic, or rather "systematising" botany, or the science of grouping together, and classifying the productions of this vast field of Nature's bounty. Most of our readers probably know, that in the present day there are two rival methods or systems, the artificial one of Linnæus, and the natural one of Jussieu, improved by Decandalle, Brown, Richard, and others. Both have great merit, and possess different and individual advantages; to compare and contrast them together, we consider absurd and illogical; their authors constructed them with different views, and these views have been in a great measure attained by each. The one is intended to be a sort of dictionary or vocabulary of the vegetable kingdom, wherein we may discover and ascertain any plant presented to our notice, in the same way as we examine a lexicon, or encyclopædia for an explanation of any word, or description of any object or event: such is the character of the Linnæan arrangement, at once so simple, beautiful, and applicable; the most important objection, indeed, to it is, that frequently it is too natural a system; for we find that just in proportion as the natural characters are obvious and distinct, so is the difficulty of distinguishing the genera and species perplexing and vexatious; look to the second order of the fifth class, containing the umbelliform plants; to the cruciform, or tretradynamic; and to the compound or syngynesious flowers, still the Linnæan method appears to us to be not only the best but indeed the only one, calculated to enable the young student to acquire a practical knowledge of botany; when more advanced, then let him begin to group and arrange the plants with which he is already acquainted, by their natural affinities and by their congenerate characters; and in this labour, he will find that Jussieu has effected more than any other author, and that his system is more comprehensive and correct than any previously unfolded. To attempt this, before he is thoroughly conversant with the plants themselves, would be like instructing a child in reading sentences, before he knew the letters, or explaining a mathematical problem to one who is ignorant of lines and angles. The remarks of Mr. Reid are so just and pertinent that we cannot do better than give them in his own words.

"According to the method of Jussieu, plants are grouped together, not because they may agree in the structure or number of any single organ, but because, on taking all their different characters into consideration, they are found to bear a strong resemblance to each other: and we also find that, in general, the properties of plants are similar in those which are like in their external characters. For these reasons, it is generally called the Natural System. An acquaintance with it affords a broad, comprehensive, and scientific view of the vegetable creation; and it embodies much important information regarding the structure, physiology, and properties of plants, and their mutual relations. Though this is the system which it must always be the object of botanical science to perfect, and with which it is necessary for every student of botany to be intimately acquainted; as it has not been found possible to simplify it as much as could be desired, a knowledge of the Linnean System must also be acquired. From its comparative simplicity, it is very convenient for those commencing the study; and, indeed, till within these few years, it alone was followed in this country." 158.

According to the natural system of classification, there are two grand, or primary divisions of plants, namely, into those which have flowers, sexual organs, and a seed provided with an embryo, and into such as are destitute of these organs; the former are denominated phenogamic, or cotyledonous, and the latter cryptogamic, or acotyledonous. The first of these classes is subdivided into two large sections, the monocotyledonous plants, or those which have one cotyledon, or seed-lobe; and the dicotyledonous, or those which have two opposite cotyledons, as in the pea, lupine, &c. &c., or several in a whirl, as in some of the coriferæ.

At this stage of our progress, it is interesting to mark the strongly stamped line of demarcation between these two grand families of plants; we can only allude to one particular, we mean the difference in the structure, or anatomy of their stems. In the monocotyledonous, the stems consist of—

"Bundles of vessels irregularly dispersed through cellular tissue, and covered by a thin cuticle (88). The Sugar-cane (*Saccharum officinarum*), the Lily, the Palm, and the Iris, have this kind of structure, the cellular and vascular tissues being blended together through the entire substance of the stem.

Stems of this kind are called *Endogenous*, because the new matter by which they increase in diameter is added at the centre. Their growth is carried on by means of the thick cluster of leaves by which they are terminated superiorly. From them the new matter descends into the centre of the stem, and pushes outwards the parts first formed. The upper parts of the leaves perish, having performed their functions; their bases remain, are pressed together, and form the new external part of the stem. In the middle of the crown of leaves is the terminal bud, which is next to be developed, to rise a little above the former, become a cluster of leaves, and in its turn be pushed outwards by a succeeding central bud.

The oldest and hardest part of such stems is that nearest to the circumference. The more the external parts are pressed by the descent of the new matter, the more close and compact they become, the outer parts being incapable of being much farther pushed out, and the whole being thus condensed into less bulk.

From the mode of growth in this stem it never can attain a great thickness, the new matter having to force outwards all the previously formed matter, which is every season increasing in quantity and becoming harder. They often, however, attain a very great height, as is seen in Palms, which are occasionally met with nearly 200 feet high.

Stems of this kind are found only in Monocotyledonous plants (406)." 28.

On the other hand, dicotyledonous plants are provided with exogenous stems, or stems in which the wood grows from without by a new layer being formed annually next the bark.

"It is pushed inwards, and becomes more compact by the pressure of each succeeding annual layer, till at last it becomes almost solid, the sides of the vessels and cells being squeezed close together: hence the great hardness of such trees in the centre."

"In exogenous plants, the new matter being added externally, a bark or covering is necessary to protect it, when young and tender, from the action of the atmosphere, and from external injury from other causes: hence an important office of the bark. In endogenous plants, the new matter, being added internally, is provided with an excellent covering, formed of the main substance of the plant, and has no need of a separate protecting integument. Thus the endogenous plant is one uniform tissue from the circumference to the centre." 36.

Without prosecuting the details further we shall satisfy ourselves by transcribing the table of the Jussieuan arrangement; and select the description of the natural families of plants, to enable our readers to form their own opinion of the manner in which Mr. Reid has executed his task.

### ORDER LV.—SOLANEÆ.

" CHARACTERS.—Herbs or shrubs, with alternate leaves, and flowers variously arranged; calyx monosepalous, in 5 (rarely 4) divisions, more or less deep, persistent; corolla with a plaited æstivation, rotate, funnel-shaped, or campanulate, with the limb 5-cleft, (rarely 4), regular (except in Verbascum); stamens 5, (occasionally 1 somewhat abortive), inserted in the corolla, and alternate with its segments; ovary 2-celled, with 2 many-seeded placentæ; style and stigma simple; pericarp a 2-celled 2-valved capsule (Datura is 4-celled and 4-valved), or a berry 2-celled, or with many cells from enlargements of the placenta; embryo curved, in the interior of a fleshy albumen.

Examples.—Henbane (*Hyosciamus niger*), Deadly Nightshade or Dwale (*Atropa belladonna*), Potato (*Solanum tuberosum*), Tobacco (*Nicotiana tabacum*). *Nolana* has a 5-lobed ovary, each lobe having 1 or 2 1-seeded cells. *Nicotiana multivalvis* has several cells external to the 2 central ones of the ovary.

Economical Properties.—The Potato is the fleshy tuber which grows on the roots or subterraneous branches of *Solanum tuberosum*. It consists almost entirely of a nutritious fecula, but is said to contain an acrid or narcotic principle; this, however, is in a very small proportion, and is dissipated by heat, as in boiling or roasting. The tubercles of *S. montanum* and *S. Venezuelæ* are of a similar nature.—*Richard.* The same author informs us, that in some countries the leaves of *S. nigrum* are boiled and eaten in the same manner as Spinach, and that the fruit is also much used in some places. The fruit of the Egg-plant (*Solanum melongena* or *S. esculentum*) is much used as an article of food in the West Indies, and in some provinces in France. The fruit of *S. lycopersicum* (Tomato or Love-apple) is frequently used for sauces; and the fruits of other species of Solanum, belonging to the Tomato section, are eatable. All these, however, are exposed to heat before being eaten. 'It is stated that the poisonous species derive their properties from the presence of a pulpy matter which surrounds the seeds; and that the wholesome kinds are destitute of this pulp, their fruit consisting only of what botanists call the Sarcocarp; that is to say, the centre of the rind in a more or less succulent state.'—*Lindley.* The dried berries and seeds of *Capsicum annuum* are known by the name of Cayenne Pepper; they are hot, pungent, and aromatic, and are used as a condiment. *C. frutescens* and *C. baccatum* have similar properties. The fruit of the Winter Cherry (*Physalis alkekengi*) is also used as a condiment.

Medicinal Properties.—The general character of this family, in a medicinal point of view, is narcotic. The root and leaves of *Atropa belladonna* are powerfully narcotic. Mr. Brandes discovered in this plant an alkali (*Atropia*), on which its narcotic properties depend, so extremely powerful, that the utmost caution is required in experimenting with it. The chief use of Belladonna is to dilate the pupil before the operation for cataract: abroad, it is used in hooping-cough. The herb and seeds of Henbane (*Hyoscyamus niger*) are also narcotic, and used in the same way as opium, where the use of the latter is inadmissible; the plant contains a peculiar alkali (*Hyoscyama*). *H. albus* and *H. aureus* have similar properties. The extreme twigs of Woody Nightshade or Bitter-Sweet (*Solanum dulcamara*) are narcotic, diaphoretic, and diurectic, but little used: an excellent bitter and tonic, said to be nearly equal to that of Cinchona, is obtained from the *Solanum pseudo-quina*. Solanum also contains a vegetable alkali (*Solanine*). The herb and seeds of Thorn Apple (*Datura stramonium*) are of a somewhat similar nature; smoking the plant has been recommended during the paroxysm of asthma. Mr. Brandes has found in this plant a vegetable alkali (*Daturine*), on which its properties seem to depend. The leaves of the Tobacco plant (*Nicotiana tabacum*) are narcotic, cathartic, emetic, diuretic, or errhine, according to the mode in which they are employed. Their use in the form of snuff or for smoking, is well known. The fruit of *Capsicum annuum* is an active stimulant and carminative, and is said to be destitute of any narcotic property. The leaves of the Mullein (*Verbascum thapsus*) are gently anodyne and emollient. The fruit of the Winter Cherry (*Physalis alkekengi*) is diuretic.

*Officinal Plants.*

| | |
|---|---|
| Atropa belladonna. | Nicotiana tabacum. |
| Solanum dulcamara. | Verbascum thapsus. |
| Hyoscyamus niger. | Capsicum annuum. |
| Datura Stramonium. | |

POISONOUS PROPERTIES.—All the plants of the above list, except the two last and perhaps *Solanum dulcamara,* are violent narcotic poisons. The Mandragore, a powerful poison, is a species of Atropa (*A. Mandragora*) : the root is the most dangerous part of the plant, but the fruit is also poisonous. The berries of *A. belladonna* have sometimes proved fatal to children. The volatile oil obtained from the leaves of Tobacco is a most virulent poison, and used by the Hottentots to poison snakes : its effects, when applied to the tongue, are almost instantaneous." 266.

---

